# Robot-Aided Motion Analysis in Neurorehabilitation: Benefits and Challenges

**DOI:** 10.3390/diagnostics13233561

**Published:** 2023-11-29

**Authors:** Mirjam Bonanno, Rocco Salvatore Calabrò

**Affiliations:** IRCCS Centro Neurolesi Bonino Pulejo, Cda Casazza, S.S. 113, 98124 Messina, Italy; roccos.calabro@irccsme.it

**Keywords:** robot-aided motion analysis, objective motor assessment, biomechanics, neurorehabilitation

## Abstract

In the neurorehabilitation field, robot-aided motion analysis (R-AMA) could be helpful for two main reasons: (1) it allows the registration and monitoring of patients’ motion parameters in a more accurate way than clinical scales (clinical purpose), and (2) the multitude of data produced using R-AMA can be used to build machine learning algorithms, detecting prognostic and predictive factors for better motor outcomes (research purpose). Despite their potential in clinical settings, robotic assessment tools have not gained widespread clinical acceptance. Some barriers remain to their clinical adoption, such as their reliability and validity compared to the existing standardized scales. In this narrative review, we sought to investigate the usefulness of R-AMA systems in patients affected by neurological disorders. We found that the most used R-AMA tools are the Lokomat (an exoskeleton device used for gait and balance rehabilitation) and the Armeo (both Power and Spring, used for the rehabilitation of upper limb impairment). The motion analysis provided by these robotic devices was used to tailor rehabilitation sessions based on the objective quantification of patients’ functional abilities. Spinal cord injury and stroke patients were the most investigated individuals with these common exoskeletons. Research on the use of robotics as an assessment tool should be fostered, taking into account the biomechanical parameters able to predict the accuracy of movements.

## 1. Introduction

In the field of neurorehabilitation, innovative technologies, such as robotic devices, have been widely used to treat and evaluate patients affected by motor impairment due to different neurological disorders (e.g., stroke, multiple sclerosis (MS), and spinal cord injury (SCI)) [1]. Compared with conventional rehabilitation approaches, robotic-assisted therapy (RAT) may have some advantages, including (i) guaranteeing repetitive, intensive, and task-oriented rehabilitation; (ii) reducing the physical burden on clinical therapists, giving them the possibility to treat more patients simultaneously; and (iii) quantitatively and objectively assessing patients’ motor performance over time [2,3]. In particular, objective assessment of motor performance is a fundamental issue in neurorehabilitation [4]. In fact, clinical scales are still widely used in hospital settings, despite their validity and reliability being under debate. Robot-aided motion analysis (R-AMA) could be helpful for two main reasons: (i) it allows the registration and monitoring of patients’ motion parameters in a more accurate way than clinical scales (clinical purpose), and (ii) the multitude of data produced using R-AMA can be used to build machine learning algorithms, detecting prognostic and predictive factors for better motor outcomes (research purpose). Specifically, motion analysis refers to the recording of three-dimensional movements of human body segments and the subsequent computation of meaningful parameters that describe human movement from raw kinematic parameters [5,6]. Motion analysis is commonly carried out through wearable and non-wearable sensors that are able to detect biomechanical parameters of movements [7]. Similarly, robotic devices, both end effectors and exoskeletons, through specific sensors, could allow the detection of passive or active range of motion, movement accuracy, and planning [8]. For example, Maggioni et al. [9] examined the possibilities of assessing lower extremity function using robots, with parameters such as range of motion (RoM), muscle strength, and proprioception. In fact, the Lokomat (which is a tethered exoskeleton) was used to assess joint position sense (i.e., proprioception) in patients with incomplete spinal cord injury. Despite their potential in clinical settings, robotic assessment tools have not gained widespread clinical acceptance. Some barriers to and doubts about their clinical adoption remain, such as their reliability and validity compared to the existing standardized scales and motion analysis.

In this narrative review, we aimed to investigate the usefulness of R-AMA systems in patients affected by neurological disorders.

## 2. Methods

Given the narrative nature of the paper, we only described the most relevant papers on the issue by searching for them on PubMed, IEEE Xplore, and Scopus, considering the period from 2010 to 2023. We chose this period because this past decade has witnessed the implementation of robotic devices in the neurorehabilitation field. To select evidence, we used the following keywords: “robotic device” OR “exoskeleton” AND “motor assessment” OR “biomechanical assessment” OR “biomechanical parameters” OR “lower limb assessment” OR “upper limb assessment” OR “motion analysis.” Since this is a narrative review, we included the most relevant pilot studies, observational studies, randomized controlled trials, case–control studies, and systematic reviews, considering also the references of the selected articles, including only English papers. Each article was evaluated through the title, abstract, text, and scientific validity [10].

## 3. Motion Analysis and Its Biomechanical Contribution to Accuracy Prediction

Motion analysis involves registering the three-dimensional movements of human body segments and then calculating biomechanical parameters that describe human movement [11]. The modeling of human motion can be studied from different perspectives. For this purpose, various approaches are used to derive mathematical expressions that describe human motion. Newton’s equations of motion are the fundamental tools for understanding the cause–effect relationship between the forces acting on a system and the resulting motion [12]. However, applying them to complex systems, such as human locomotion, which involve a large number of degrees of freedom, requires formulating and solving multiple equations, leading to high computational costs. The Euler–Lagrange method is used in multibody systems because it analyzes the entire system without studying the reaction and contact forces between the elements that comprise the system. This equation allows for the study of human motion by focusing solely on the mechanical energy of the system. The knowledge of motion equations allows researchers to identify problems and design mechanisms that seek to recognize or recover human movements [13]. Nowadays, motion analysis has evolved substantially in parallel with technological advancements, encompassing various applications, such as clinical gait analysis and 3D biomechanical modeling [14]. Biomechanical motion analysis is generally based on two types of models: the multibody model and the finite element model. The first type consists of a set of rigid or flexible bodies connected by joints, while the second type of motion analysis reconstructs internal strain, stress, or deformation in flexible bodies based on continuum mechanics theories [15,16].

Within a rehabilitation setting, quantitative analysis of human body kinematics is a powerful tool that has been used to understand the different biomechanical patterns of both healthy and pathological individuals [17]. Recently, biomechanical tools have also been developed, ranging from simple manual annotation of images to marker-based optical trackers and inertial sensor-based systems. Nowadays, motion analysis can be performed using marker-less systems that use sophisticated human body models, computer vision, and machine learning algorithms [17]. Biomechanical parameters that are considered during motion analysis include kinematic and kinetic parameters [18,19]. In particular, kinematic parameters [20] include the spatial and temporal aspects of movement. These parameters describe (a) the “static” direction during point-to-point movements; (b) the continuous change of position, speed, and acceleration, which can be further subdivided into its amplitude and direction components; or (c) combinations of these, such as movement trajectories.

## 4. Robotic Devices for Upper Limb Measurement 

Kinematic (e.g., position, velocity, and acceleration) and kinetic (e.g., force, joint torque, and muscle activity) data are acquired from sensors affixed to robotic and passive mechanical devices to measure biomechanical aspects of upper extremities [21,22,23,24,25,26,27,28] (see more in Table 1).

**Figure 1:**
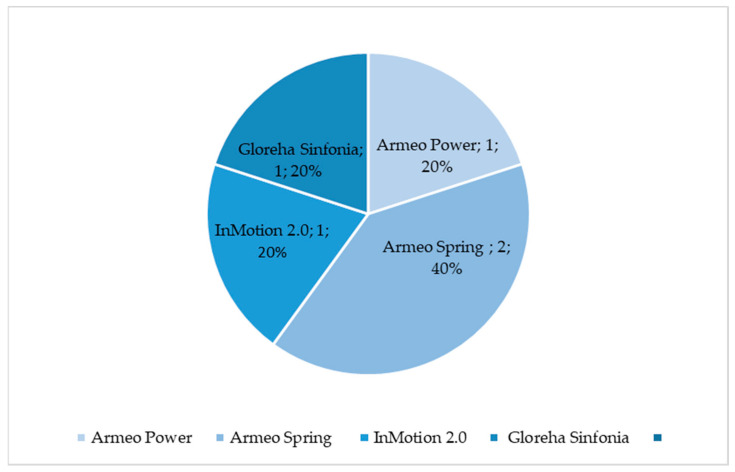
Percentage of selected articles reported in Table 1 dealing with upper limb robotic-aided motion analysis.

These kinds of measures are commonly registered in post-stroke patients, who may present unilateral hemiplegic involvement. However, the percentage of studies dealing with R-AMA for upper limbs is still poor. It seems that the Armeo^®^Spring was the most used for this issue, followed by the Armeo^®^Power, InMotion 2.0, and Gloreha Sinfonia, as reported in Figure 1. 

For example, one of the most used robotic devices in post-stroke neurorehabilitation is the Armeo^®^Power, an exoskeleton for upper limb training. Its efficacy in improving functional outcomes is already demonstrated in the literature [34,35]; however, few authors have investigated its role in assessing upper limb functions.

Specifically, this robotic device can evaluate specific kinematic parameters [36], as reported in Table 1. In addition, the Armeo^®^Power evaluates the range of joint movement, which is expressed in degrees, and the force of muscles, which is expressed in Newton meters (Nm). According to Galeoto et al. [29], the Armeo^®^Power can be considered an objective robotic tool compared to the Fugl–Meyer for upper limb (FM-UL) clinical scale items. The FM-UL clinical scale is the most used and reliable scale to assess motor functions, joint range of motion, joint pain, dysmetria, and tremor in post-stroke patients [37]. The authors found strong correlations between flexion synergy (forearm supination and elbow flexion) and results measured with the Armeo^®^Power. This suggests that the Armeo^®^Power is more accurate than the FM-UL clinical scale in evaluating upper limb movements [29].

Other researchers have also evaluated the motor function of stroke patients using robotic devices and measuring upper limb biomechanical features, such as movement velocity, accuracy, and smoothness in active training [30,31]. Merlo et al. [30] used the Armeo^®^Spring to conduct these measurements. To obtain objective data on upper limb functions, the Armeo^®^Spring calculates a set of numerical indices based on the 3D endpoint trajectory during the “vertical capture” task. The patient receives visual feedback of their hand position through a display, which is used to facilitate rehabilitation exercises. Indeed, the derived indices (movement velocity, accuracy, and smoothness) are easy to share with clinicians because they describe the motor impairment of the upper limb [28].

For example, the loss of movement accuracy can be related to a reduction in sensibility, whereas the decrease in velocity refers to paresis/paralysis, and the loss of smoothness refers to an abnormal muscle tone (spasticity) [38]. However, before implementing them in clinical practice, these indices must be validated by comparing them with other clinical scales. In their study, Longhi et al. [31] analyzed three aspects of upper limb (UL) evaluation. First, they examined the ability of the Armeo^®^Spring to distinguish between stroke patients and healthy subjects. Second, they assessed the validity of the indices used to measure movement. Lastly, they investigated the concurrent validity of these indices by comparing them with the Wolf Motor Function test, a clinically validated scale for assessing UL motor function. The authors’ results confirmed the construct validity of the three indices, which is consistent with the findings of Merlo et al. [30]. This suggests that the Armeo^®^Spring can be a promising tool for objectively assessing UL motor skills. In addition, Goffredo et al. [32] performed a kinematic evaluation of the upper limb in post-stroke patients using the end effector InMotion 2.0.

The kinematic parameters were calculated from the trajectories recorded by the robot, starting from the central target and extending to the peripheral targets in various directions. The kinematic parameters described by the authors [32] refer to the functional abilities of the UL. However, the Armeo^®^Power and the Armeo^®^Spring cannot perform hand motion analysis due to their biomechanical architecture. To this aim, Cordella et al. [33] conducted a quantitative and objective assessment of hand movement in post-stroke patients using the Gloreha Sinfonia. The Gloreha Sinfonia is a robotic glove used to train hand motor functions, focusing on the recovery of range of motion [33]. Once calibrated, this glove allows an objective assessment of motor performance. In particular, the results of the authors [33] demonstrated that the Gloreha Sinfonia can measure angular values from bending sensors embedded in the glove.

Another concern that should be considered in clinical practice is the objective evaluation of spasticity. The Modified Ashworth Scale (MAS) is, in fact, the most commonly used clinical tool for assessing spasticity. However, it does have several limitations [39]. Indeed, de-la-Torre et al. [38] in their systematic review found that R-AMA based on data capture is effective for evaluating spasticity. However, it should be noted that cutting-edge algorithms provide a more predictive and analytical measure than the only variation between the original and the final status obtained from clinical scales [38]. Moreover, some authors [40] have evaluated muscle synergies in post-stroke patients using a robotic device. Muscle synergy specifically refers to the coordinated activation of both joints and muscles in order to execute purposeful movements [41,42]. Post-stroke patients tend to activate abnormal muscle synergies due to brain lesions in the corticospinal tract, which are further enhanced by hyperreflexia. This aspect is fundamental in establishing the most effective treatment for patients in the clinical rehabilitation setting. In this vein, Kung et al. [40] found that robotic devices, such as end effectors, can be used for long-term evaluation of muscle synergies.

They registered kinematic, kinetic, and electromyographic (EMG) signals during the tracking movement in order to develop biomechanical indices for evaluating muscle synergies. In fact, their results revealed that abnormal synergies can be assessed through two tracking directions: D2 (contra-proximal to ipsi-lateral) and D4 (left–right) [40]. Lastly, robotic devices can also measure muscle strength, as suggested by Toigo et al. [43]. In particular, the term “muscle strength” refers to force, moment, or power [43]. Robotic devices, including exoskeletons and end effectors, are equipped with force sensors for quantifying the interaction forces between the device and the patient [44]. These devices record raw sensor data on force during functional movements, enabling the extraction of valuable data detecting abnormal muscle synergies [43]. However, misalignments with the device and variations in the rotational axis of a joint can distort the results. Moreover, all kinematic and kinetic movement parameters are represented to some extent in the sensorimotor cortex. Distal movements of the hand, including movement direction and trajectories, can be discriminated in the sensorimotor cortex. This ability has potential applications in brain–computer interface technology [21].

## 5. Robotic Device for Lower Limb Assessment

Walking recovery in neurological patients is one of the most important goals planned by therapists [45]. In order to maximize the recovery of the walking function, it is important to define a personalized rehabilitation treatment, in addition to an accurate assessment to monitor patients’ progress. In fact, both clinical and instrumental tools already exist to perform an accurate analysis of motion [45]. However, if the assessment protocol takes too much time to perform, clinicians and therapists may be reluctant to adopt them. A possible solution could involve the use of robotic devices in which the patient would undergo both training and assessment. In this study, Imoto et al. [46] used a novel gait training robot known as WelWalk WW-2000. This robot enables the adjustment of various gait parameters (such as time and mechanical assistance load) during the training session. The robot is equipped with sensors and a markerless motion capture system to detect altered gait patterns in stroke patients. This system can evaluate individuals’ gait patterns and provide tailored rehabilitation gait training [46]. Generally, the objective assessment of the lower limb should consider the simultaneous measurement of joint angles, spatial and temporal parameters of gait, muscle strength, proprioception, and spasticity and/or muscle stiffness [47] (see Table 2).

**Figure 2:**
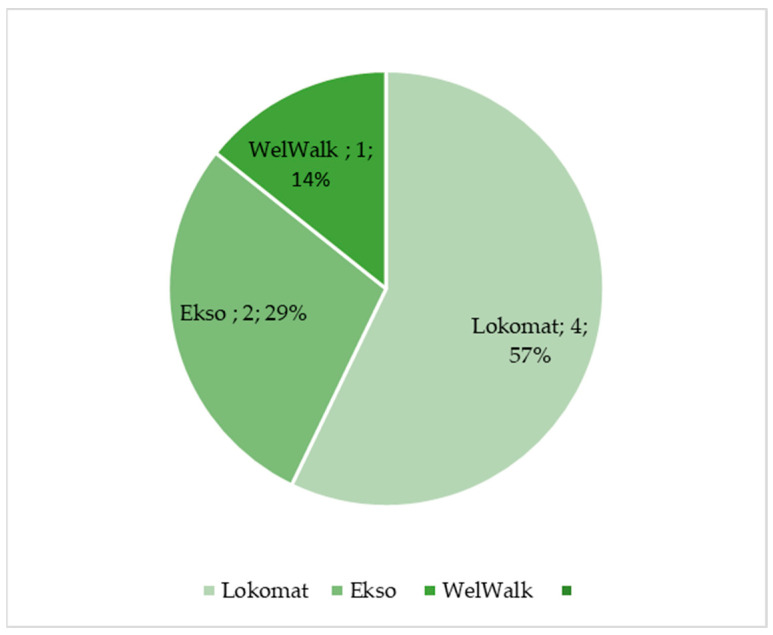
Percentage of selected articles reported in Table 2 dealing with lower limb robotic-aided motion analysis.

The Lokomat, which is a tethered exoskeleton, is one of most used robotic devices for gait training and for motion analysis in neurological disorders. In fact, 57% of the selected papers reported the use of the Lokomat in performing R-AMA, followed by Ekso and the WelWalk, as reported in Figure 2.

According to a systematic review [53], the Lokomat seems to be most suitable for the motion analysis of lower limbs. Maggioni et al. [54] used the Lokomat to perform a type of gait analysis, also adding force sensors and potentiometers. The authors successfully developed and tested a novel specific algorithm to assess walking through the Lokomat. Indeed, the Lokomat was used to calculate joint angles, assuming that those measured by the exoskeleton also corresponded to the human angles [54]. Mercado et al. [55] calculated joint angles in healthy subjects using the Denavit–Hartenberg notation and the Euler–Lagrange approach to process video recordings of movement. Another study [48] investigates the use of Ekso-GT, an overground exoskeleton, to assess gait parameters, such as stride time, stride length, gait speed, and gait events. Although Ekso does not provide a comprehensive report of gait parameters, these parameters and measurements can be derived from other calculations made by the exoskeleton. This allows for an accurate assessment of gait during training using mathematical models. In addition, exoskeletons, like Ekso, can be integrated with surface electromyography (sEMG) signals to monitor muscle synergies and muscular patterns during walking. According to a systematic review [56], the rectus femoris and vastus lateralis are the most frequently recorded muscles during gait. Indeed, the posterior calf muscles, which play a role in ankle and foot movement, have been less studied during gait training, despite their importance in the gait cycle. Similarly, Afzal et al. [49] investigated muscle synergies in patients with MS who were wearing an exoskeleton. EMG signals were recorded from seven muscles, including the vastus medialis, rectus femoris, biceps femoris, semitendinosus, soleus, medial gastrocnemius, and tibialis anterior muscles. The authors demonstrated that exoskeleton assistance does not alter the existing muscle synergies but it can induce a modification in neural commands [49].

Another point to consider is the evaluation of proprioception provided by robotic devices. Three studies [50,51,52] in spinal cord patients have addressed the evaluation of proprioception or kinesthesia using the Lokomat. In fact, the Lokomat is equipped with position sensors that are able to determine joint angles. For proprioception, the authors considered the difference between the target position and the achieved position for evaluation purposes [50,51]. Another author [52] evaluated kinesthesia by passively moving the lower limb in a specific direction while patients were wearing the exoskeleton.

## 6. Discussion

In this narrative review, we found that robotic devices may be used to assess motor behavior in patients with neurological disorders. Indeed, according to the few available studies, two main exoskeletons, namely the Lokomat and the Armeo^®^Spring, R-AMA may provide clinicians and researchers with reliable and more objective data regarding motion analysis of the lower and upper limbs, respectively [30,31,50,51,52,53,54]. In addition, upper limb R-AMA was tested only in post-stroke patients [29,30,31,33,36,37], while other neurological disorders were excluded. This issue could be related to the fact that the motor symptoms of other neurological pathologies, specifically those related to MS, are often complicated by ataxia or extrapyramidal signs. These complications have a negative impact on motion analysis [45]. Indeed, post-stroke patients manifest moderate-to-severe upper limb sequalae (mainly weakness with hypotonia in the acute phase) due to damage in the cortico-spinal tract [57]. Similarly, lower limb R-AMA was mostly performed on patients with SCI [50,51,52], who are characterized by severe lower limb motor impairments, mostly due to the traumatic interruption of central nervous pathways. Given that R-AMA was performed only in patients with moderate-to-severe motor impairment, future studies should take into account other levels of severity, as well as consider other pathologies. (e.g., MS, PD, and traumatic brain injury).

### 6.1. Benefits of Robotic-Aided Motion Analysis

Compared with conventional assessment methods, such as clinical scales or tests administered by physiotherapists and/or physicians, R-AMA offers several advantages. It can provide tri-axial measurements, analyze the patient’s limb trajectory, accurately register spatial-temporal parameters of movement, and collect a large amount of data. Altogether, these elements allow for personalization of the rehabilitation path according to patients’ needs. This personalized approach can be used to create a tailored patient profile, which includes a precise physiotherapy program. This program considers both traditional and cutting-edge devices for treatment. In recent years, the concept of personalized treatment has gained significant traction in various medical fields [58], including neurology and rehabilitation.

**Figure 3:**
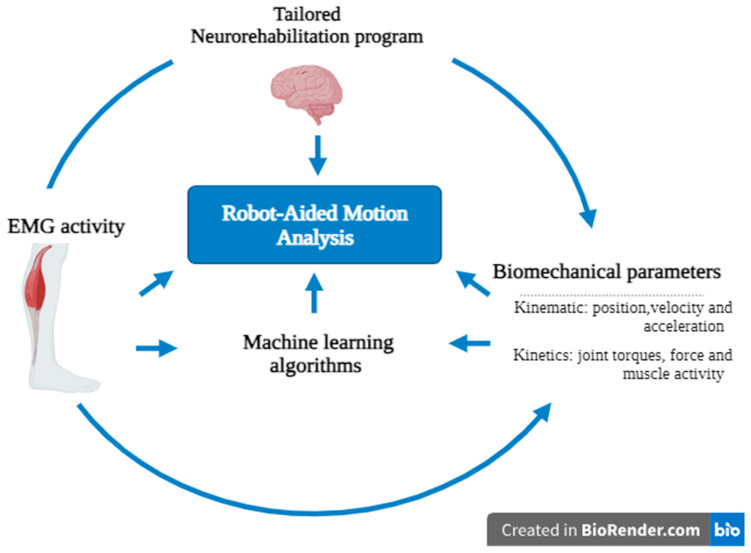
Graphic illustration showing the theoretical usefulness of robot-aided motion analysis in the field of neurorehabilitation. The figure was created with Biorender.com.

In this vein, the so-called “rehabilomics” sheds some light on the role of biomarkers in the clinical and rehabilitation setting [59]. This approach has primarily focused on the biological field, including proteomics, genomics, metabolomics, and other related areas. However, the kinematics and electrophysiological indicators can be considered biomarkers, as suggested by Garro et al. [60]. Indeed, the development of biomarkers based on the models of motor control mechanisms may be useful in a clinical context to understand healthy functions, disability, and rehabilitation progress. In this way, R-AMA can conduct a neuromechanical assessment, which examines the connection between neurological pathology and biomechanical issues [61] (Figure 3).

Legend: R-AMA could be useful to personalize neurorehabilitation programs, thanks to both biomarkers provided by EMG biosignals (on the left) and biomechanical parameters (on the right), including kinematic and kinetics. In the end, the great quantity of data obtained through R-AMA could be further used for MLA for individuating motor biomarkers involved in recovery prediction.

### 6.2. Challenges of Robotic-Aided Motion Analysis

To date, research on robotic devices has primarily focused on neuromotor training and recovery in patients with neurological disorders, without considering the potential role of these devices in objectively evaluating movement. However, physicians frequently criticize these technical solutions, claiming that the outcome measures offered by robotic devices are too abstract, do not translate into practical function, and lack ecological validity. 

An important point that should be addressed is that robotic devices may require a lengthy setup time and the support of technical staff to operate. In the clinical setting, the physiotherapist has 30–60 min of rehabilitation treatment for each patient, and this could further discourage the use of robotic devices in clinical practice. Additionally, robotic devices, especially exoskeletons, must be perfectly aligned with the user to avoid undesired interaction forces [62]. These forces can result in an uncomfortable and unsafe human–robot interaction in the case of high forces or torques. Solutions to address misalignment of the joint’s axes can include soft exoskeletons, which are constructed from soft textiles or elastomers. These exoskeletons offer greater user compliance compared to rigid robotic orthoses [63]. However, robotic devices are not available in all rehabilitation centers due to their costs, maintenance requirements, and the need for additional staff [64]. These may be some reasons why robot-based assessments have not yet been integrated into clinical practice on a large scale.

However, recent technological developments in the field of wearable devices, such as accelerometers and inertial sensors, have the advantage of providing objective motion analysis as low-cost and easy-to-use tools, as opposed to robotic devices [17,45]. In this sense, professional engineers should be encouraged to develop assessment technologies that are not constrained by practical limitations and administrative burdens. In our opinion, we must identify and overcome the barriers that prevent the translation of robotic evaluations to clinical application.

### 6.3. Future Perspectives: Combined Approaches and Beyond

The selection of motion biomarkers predicting recovery remains an open and under-debate question. According to Amrani El Yaakoubi et al. [56], EMG and biomechanical parameters together, including both kinetic and kinematic factors, are the most used predictors for lower limb movement. EMG is, in fact, sensitive to neuromuscular changes, particularly in post-stroke patients. The most common surface EMG analyses are time-domain and frequency-domain analyses. Specifically, among frequency-domain analyses, the mean frequency and median frequency are the most effective to assess muscle fatigue in post-stroke patients [65]. Hussain et al. [66] found that a machine learning neural network model based on EMG frequency domains has a high level of accuracy. However, the muscles that contribute the most to kinetic and kinematic prediction cannot currently be defined due to the heterogeneity of the results from the studies. In contrast, the kinematic assessment of the upper limb mainly includes the smoothness of the trajectory, as suggested by various authors [66,67]. Scano et al. [68] identified that post-stroke patients exhibit lower smoothness of trajectory, indicating difficulty in controlling the upper limb during multi-joint movements. Also, the authors found that elbow and shoulder joints showed a limited ROM, likely due to altered postural accommodation. In this view, analyzing EMG signals during upper limb functional activities with or without exoskeletons could be a future objective to achieve. Moreover, other biosignals, like EEG, can be used to control robotic devices through the brain–computer interface (BCI). An EEG-based brain-controlled robot is a robotic device that uses an EEG-based BCI to receive control commands from its user [69]. In the field of neurorehabilitation, EEG-based brain-controlled assistive robots are divided into manipulators and mobiles. Brain-controlled manipulators operate under direct BCI control, with user commands being sent to the robots. This is done without the need for additional assistance from robot intelligence elements [70]. In contrast, brain-controlled mobiles operate under shared BCI control, which involves combining a BCI system with an intelligent controller. Robots of this type are safer, less tiring for their users, and more accurate in interpreting and executing their commands [71,72]. Therefore, future developments in rehabilitation robotics should enable physicians to choose the most appropriate biomechanical parameters according to an individual patient’s specific requirements. Future technological advancements in the assessment of motor performance should consider kinematic, EMG, and EEG signals. This aspect could be crucial in understanding how the brain’s sensorimotor cortex encodes movements to achieve optimal neural control of motor performance. It also enables the differentiation between healthy and pathological characteristics. Hence, in order to guide the development of future robotic-based assessment tools, it is essential to foster multidisciplinary collaboration between clinical professionals (such as neurologists, physiatrists, and physiotherapists) and biomedical engineers.

## 7. Conclusions

In conclusion, the utility of R-AMA for both clinical and research purposes is still a subject of debate, although some promising findings have been reported regarding the effectiveness of the Lokomat and the Armeo. The motion analysis provided by these robotic devices is used to customize rehabilitation sessions, relying on the objective quantification of patients’ functional abilities. It should be considered that clinical scales and tests used to monitor motor recovery in neurological patients are less accurate than motion analysis conducted by robotic devices. Next, research on the use of robotics and assessment tools should be encouraged. Future studies should be oriented toward two different frontiers: (1) understanding the most useful biomechanical parameters that can predict the accuracy of movements and (2) validating robotic device assessments for clinical purposes.

## Figures and Tables

**Table 1:** Studies about upper limb robotic-aided motion analysis performed in neurological disorders.

Reference No.	Robotic Device	Description	Usefulness of Robot-Aided Motion Analysis
[29]	Armeo^®^Power (Hocoma AG, Switzerland)	The Armeo^®^Power is a 6-degrees-of-freedom exoskeleton for upper limb rehabilitation.	Useful tool for the objective evaluation of upper limbs in post-stroke patients. The kinetic parameters of the motion analysis included kinetic parameters of the shoulder (flexion–extension, abduction and adduction, internal and external rotation), of the elbow (flexion–extension, prone–supination), of the wrist (flexion–extension), and of the hand (opening and closing). The values deriving from the valuation of the articular range were expressed in degrees; the values deriving from the evaluation of the force were expressed in Newton meters (Nm).
[30]	Armeo^®^Spring (Hocoma AG, Switzerland)	The Armeo^®^Spring device is an exoskeleton for upper limb rehabilitation. It is equipped with 7 goniometers and 1 pressure sensor, which permits free 3D arm movement. At the end of the robotic arm, there is a handle, which contains a pressure sensor, measuring the grip force.	The authors used the Armeo^®^Spring device to conduct a quantitative assessment of the precision, speed, and smoothness of upper limb motion. Among the several measures, the hand path ratio is the ratio between the actual path in the horizontal plane and the shortest-possible path, which reflects movement efficiency. The mean velocity and the number of peaks in the velocity profile were also assessed. Additionally, the normalized jerk (Norm Jerk), a measure of trajectory smoothness, was analyzed.
[31]	Armeo^®^Spring (Hocoma AG, Switzerland)	As described before	The Armeo^®^Spring was used to assess movement accuracy by measuring the hand path ratio, the mean velocity, and the number of peaks in the velocity profile. The authors concluded that the device should be integrated into the clinical evaluation of upper limb functions in post-stroke patients.
[32]	InMotion 2.0 (Bionik Laboratories, Watertown, MA, USA)	The InMotion 2.0 device is an end effector in which the subject moves their arm from a central target to 8 peripheral targets.	The authors assessed kinematic parameters of the upper limb, including elbow extension and shoulder flexion, abduction and external rotation of the shoulder, elbow flexion and shoulder extension, and adduction and internal rotation of the shoulder. These parameters, calculated at baseline, can assist clinicians in defining a rehabilitation program for post-stroke patients.
[33]	Gloreha Sinfonia (Idrogenet, Lumezzane BS, Italy –)	Gloreha Sinfonia is a robotic glove for hand rehabilitation to maintain range of motion (i.e., the flexion angle excursion of the finger metacarpophalangeal joints) of the patient’s hand.	The authors objectively evaluated hand movements using the Gloreha Sinfonia glove in order to customize rehabilitation sessions according to patients’ motor abilities. The angular values of the joints were assessed using bending sensors embedded in the glove.

**Table 2:** Studies about lower limb robotic-aided motion analysis performed on neurological patients.

Reference No.	Robotic Device	Description	Usefulness of Robot-Aided Motion Analysis
[46]	WelWalk (WW-2000, Toyota Motor Corporation, Aichi, Japan)	Knee-ankle-foot robot, low floor treadmill, safety suspension device for body weight support, monitor for patient use, 3D sensor, and control panel	Three-dimensional joint positions, lower limb tilt, and knee joint angle were recorded during a task using a 3D sensor, an inertial sensor, and a knee angle sensor. Two-dimensional joint positions collected using skeletal tracking software (VisionPose^®^, NEXT-SYSTEM Co., Ltd., Fukuoka, Japan) and depth data from the 3D sensor were used to estimate the three-dimensional coordinates of the joint positions. Bilateral hip, knee, ankle, and shoulder joints, as well as the midpoints of the shoulder and hip joints, were the predicted locations of the 3D joints. This objective gait analysis can be useful for individuals with hemiparetic stroke, as it provides individually tailored gait training based on these assessments.
[48]	Ekso (Ekso Bionics, San Rafael, CA 94901, USA)	Ekso a wearable unthethered exoskeleton. Motors power the hip and knee joints and all motion are started either through specific patient actions or the use of an external controller.	The authors conducted a comprehensive assessment by utilizing both kinematic and kinetic parameters, as well as EEG registrations, in patients with Parkinson’s disease. In this way, clinicians can personalize the rehabilitation treatment with a device that could increase the treatment intensity and dose without burdening therapists.
[49]	Ekso (Ekso Bionics, San Rafael, CA 94901, USA)	As described before	Muscle synergies and activation profiles were extracted using non-negative matrix factorization. The authors’ findings provided insights into the potential underlying mechanism for improving gait functions through exoskeleton-assisted locomotor training.
[50]	Lokomat (Hocoma AG, Switzerland)	The Lokomat is a robotic tethered exoskeleton with active hip–knee actuation and passive ankle control during the swing phase, in addition to a variable level of assistance.	The Lokomat was used to assess proprioception, which provides information about static position and movement sense, using custom software to measure joint position sense in the hip and knee. The authors demonstrated the usefulness of the Lokomat in measuring proprioception in SCI patients.
[51]	Lokomat (Hocoma AG, Switzerland)	As described before	The authors proved the Lokomat’s usefulness in objectively assessing proprioception at the hip and knee in people with SCI.
[52]	Lokomat (Hocoma AG, Switzerland)	As described before	Since lower limb kinesthesia deficits are common in SCI patients, the authors demonstrated that the Lokomat can serve as a valid and reliable robotic device for monitoring sensory function. Kinesthesia was evaluated using angular encoders of the hip and knee. During the analysis, a score was generated based on the difference between the initial angle and the final angle.

## Data Availability

Not applicable.
